# Administration of fusion cytokines induces tumor regression and systemic antitumor immunity

**DOI:** 10.1002/mco2.68

**Published:** 2021-05-04

**Authors:** Jinyu Zhang, Xuan Zhao

**Affiliations:** ^1^ Mianyi Biotech Corporation Chongqing China; ^2^ Institute for Immunology and School of Medicine Tsinghua University Beijing China

**Keywords:** cancer immunotherapy, fusion cytokine, immunocytokine, interleukin 12, interleukin 2, granulocyte macrophage colony stimulating factor

## Abstract

It is difficult to improve the curative effects of cancer immunotherapy on solid tumors. Cytokines, as powerful immune regulators, show potential in activating host antitumor immunity. We have previously found that the administration of certain cytokine combinations induces complete tumor clearance. Here, we constructed cognate fusion cytokines and evaluated their antitumor effects in various mouse tumor models. The in situ induction of the expression of the fusion cytokine IL12IL2GMCSF caused tumor eradication, including that of the tumors at advanced stages. An immune memory against unrelated syngeneic tumors was also elicited. Furthermore, flow cytometry analysis revealed that tumor‐infiltrating CD3+ cells were greatly increased in the treated tumors and were accompanied by an elevation of CD8+/CD4+ ratios. This fusion protein exhibited superior immune activating capability compared to that of cytokine mixtures, in the experiments done in vitro. We also induced tumor regression in various immunocompetent tumor models via intratumoral injection. To improve its translational potential for clinical application, a systemically‐administered immunocytokine, IL12IL2DiaNFGMCSF, was constructed by inserting a tumor‐targeting diabody in the fusion protein. This protein also displayed good immune stimulating activities in vitro. Intravenous infusion of IL12IL2DiaNFGMCSF induced tumor‐infiltrating immune cell alterations like IL12IL2GMCSF, with moderate serum IFNγ increment. Therapeutic effects were observed in the various tumor models after systemic administration of IL12IL2DiaNFGMCSF, but with slight toxicity. These results show the feasibility of developing a versatile cancer immunotherapy.

## Graphical abstract

Here, we constructed cognate fusion cytokines and evaluated their antitumor effects in various mouse tumor models. The fusion cytokines can elicit antitumor immunity through synergistic effects on the immune cells. Administration of these proteins induced tumor regression in various tumor models with slight toxicity. These results show the feasibility of developing a versatile cancer immunotherapy.

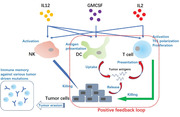

## 1 INTRODUCTION

In 2018, nearly 10 million people died from malignant tumors worldwide.^1^ The use of cancer immunotherapy has brought new hope for the treatment of these fatal diseases.^2^ Among the various emerging therapeutics, chimeric antigen receptor (CAR)‐T cell therapy and immune checkpoint inhibition are the two most promising remedies.^3^, ^4^ They both exhibit beneficial effects in the treatment of hematologic malignancies and solid tumors.^5^, ^6^, ^7^, ^8^ However, the several efforts made with respect to the study of these therapies have brought little advancement in terms of further improving therapeutic outcomes. Large amounts of clinical evidence suggest that only a few patients benefit from immune checkpoint inhibition,^9^, ^10^ wherein the antitumor response is long‐lasting. The situation is different with CAR T cell therapy. T cell infusion leads to disease remission in most patients, but with a high refractory rate.^11^, ^12^ To solve these problems, immunotherapy, in combination with other modalities, is being tested in various cancers.^13^, ^14^, ^15^, ^16^ Although combination therapies improve the outcomes in some experiments, many clinical trials have failed due to inadequate study designs derived from an incomplete understanding of the mechanisms behind tumor immunity.^17^, ^18^, ^19^

Cytokines are important molecules that regulate the immune network of the body.^20^, ^21^ They are very potent, since low concentrations of them are enough to induce strong immune reactions. Upon stimulation, immune cells secrete cytokines that influence other cells. Meanwhile, immune cells are also affected by cytokines from other cells. Cytokines also play a role in the crosstalk of immune cells, leading to specific immune responses.^22^, ^23^, ^24^ However, their mechanisms of action are similar to neutral networks, in that they are hard to understand and control. A cytokine may function differently in different immune contexts. It is necessary to consider the indications and microenvironments before using these in the treatment of diseases.

Some cytokines, alone or in combinations, have exhibited beneficial therapeutic effects in cancer therapy on experimental animals.^25^, ^26^, ^27^ However, the clinical outcomes are not satisfactory in terms of their toxicity windows. Despite the potential of cytokines to treat tumors, severe side effects restrict their clinical application.^28^, ^29^ For example, interleukin 12 (IL12) has promising antitumor activities in murine tumor models; however, the maximal tolerated dose (MTD) is only 0.5–1 μg/kg, restricting its clinical application.^30^, ^31^ Some cytokines, such as IL12 and interleukin 2 (IL2), have great potential in enhancing cytotoxic cellular immunity,^32^ which is considered necessary for tumor eradication.^33^, ^34^ Because malignant tumors confront the immune system through various mechanisms, strategies for treating cancer using these molecules require further investigation.

In a previous study, we found that some combinations of cytokines in triples exhibited unexpected antitumor activities after local administration to tumor sites, which is mediated by the activation of the immune system.^35^ Among them, the combination of IL12, IL2, and granulocyte macrophage colony stimulating factor (GMCSF) showed the highest antitumor activity. It is difficult to translate a mixture of biomacromolecules for standard pharmaceutical manufacturing and clinical usage. Here, we designed fusion proteins of these cytokines and evaluated their therapeutic effects in mouse tumor models. The study demonstrates the potential of translating this preclinical modality into therapy for malignant tumors therapying humans.

## 2 RESULTS

### 2.1 Induced expression of fusion cytokine dcIL12IL2GMCSF led to tumor regression and antitumor immunity

**FIGURE 1:**
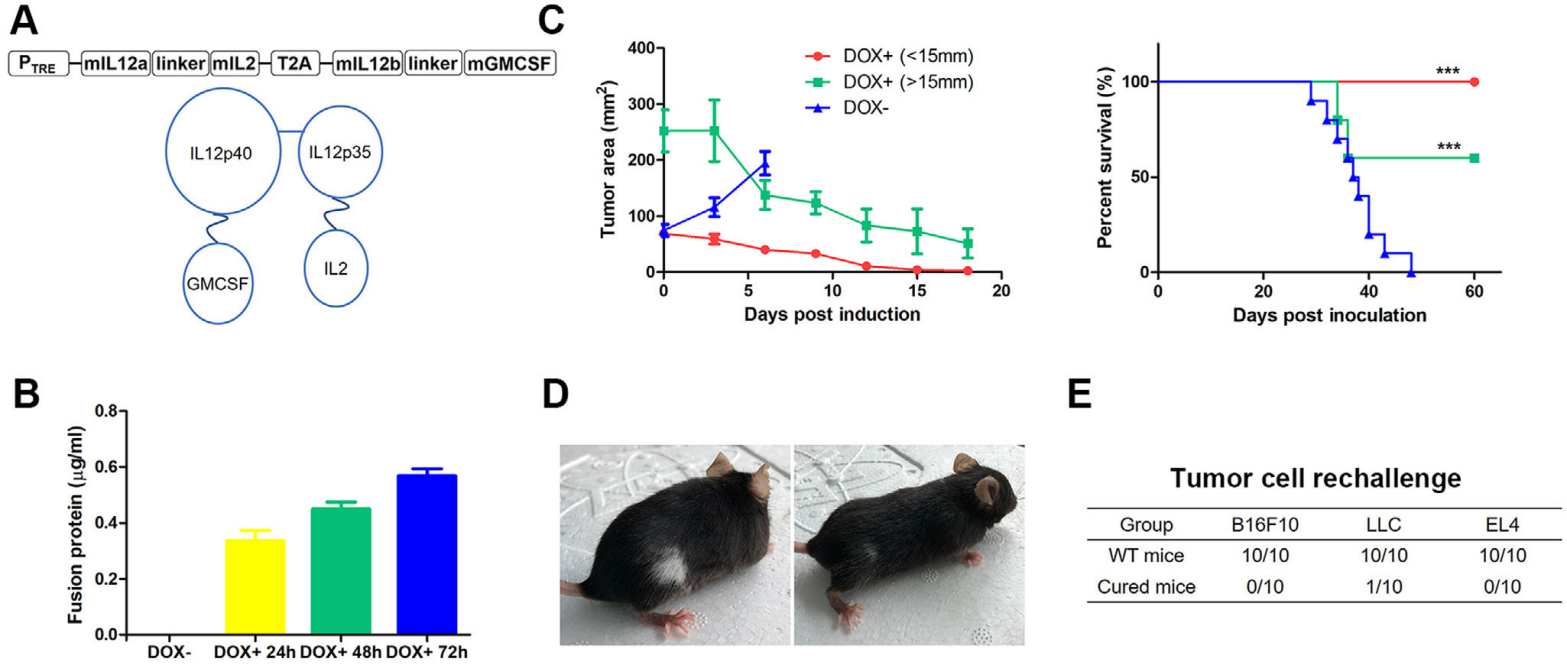
Induced expression of dcIL12IL2GMCSF led to tumor regression and antitumor immune memory. (A) Schematic representation of the dcIL12IL2GMCSF expression cassette and molecule conformation. (B) The expression of dcIL12IL2GMCSF was induced by dox addition. At different time points, the supernatants were collected and subjected to ELISA measurement of fusion cytokine levels. *n* = 3. (C) The inducible B16F10 cells were subcutaneously inoculated into the flanks of C57BL/6 mice. Dox was administered in the drinking water of the mice when the tumors reached indicated sizes. Tumor growth (*n* = 5) and overall survival (*n* = 10) were recorded. ***p < 0.001. The experiments were repeated three times. (D) Representative photos of the vitiligo that occurred in the cured mice. (E) The cured mice were subcutaneously injected with 10^5^ B16F10, 2 × 10^5^ LLC, or 5×10^5^ EL4 tumor cells 10 months after the original tumor inoculation. Age‐matched wild type mice were used as controls. The numbers in the table indicated death/inoculated mice

First, we constructed an inducible B16F10 cell line model to evaluate the antitumor capability of the fusion protein dcIL12IL2GMCSF, a heterodimer comprising two polypeptides ligated with disulfide bonds between IL12p35 and IL12p40 subunits (Figure 1A). The expression of the protein could be effectively induced by administering doxycycline (dox), at low background levels (Figure 1B). The tumor cells were subcutaneously inoculated into the flanks of mice, and induction was initiated when the tumors reached indicated sizes. The expression of dcIL12IL2GMCSF completely erased tumors that were less than 15 mm in diameter. Among the mice with large tumors that had diameters over 15 mm, 40% died within 3 days after administering dox. However, the remaining mice exhibited elimination of the large tumors and gained long‐term survival (Figure 1C). There were symptoms of vitiligo observed at the original tumor sites in the cured mice (Figure 1D). Notably, no systemic vitiligo symptoms were observed even up to 12 months after tumor regression. More than 10 months after the initial treatment, the cured mice were rechallenged with parental B16F10 cells or irrelevant syngeneic tumor cells, which include Lewis lung carcinoma (LLC) and EL4 cells. Except for one mouse in the LLC group, all the other mice completely rejected the inoculated malignant cells (Figure 1E). This indicated the existence of an immune memory against various tumor antigens, which was derived from the eradication of the primary inducible B16F10 tumors.

### 2.2 Immune cell alterations induced by dcIL12IL2GMCSF expression

**FIGURE 2:**
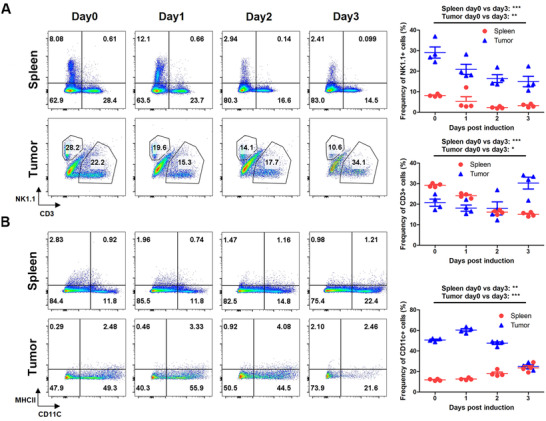
The alterations of immune cell populations during induced expression of dcIL12IL2GMCSF. The inducible B16 cells were subcutaneously inoculated into the flanks of C57BL/6 mice. At different times after dox administration, splenocytes and tumor‐infiltrating lymphocytes were isolated and subjected to flow cytometry analysis. The gated CD45+ cells were analyzed with the groups of NK1.1, CD3 (A) or CD11C, MHCII (B). The experiments were performed twice. *p < 0.05; **p < 0.01; ***p < 0.001. *n* = 4

**FIGURE 3:**
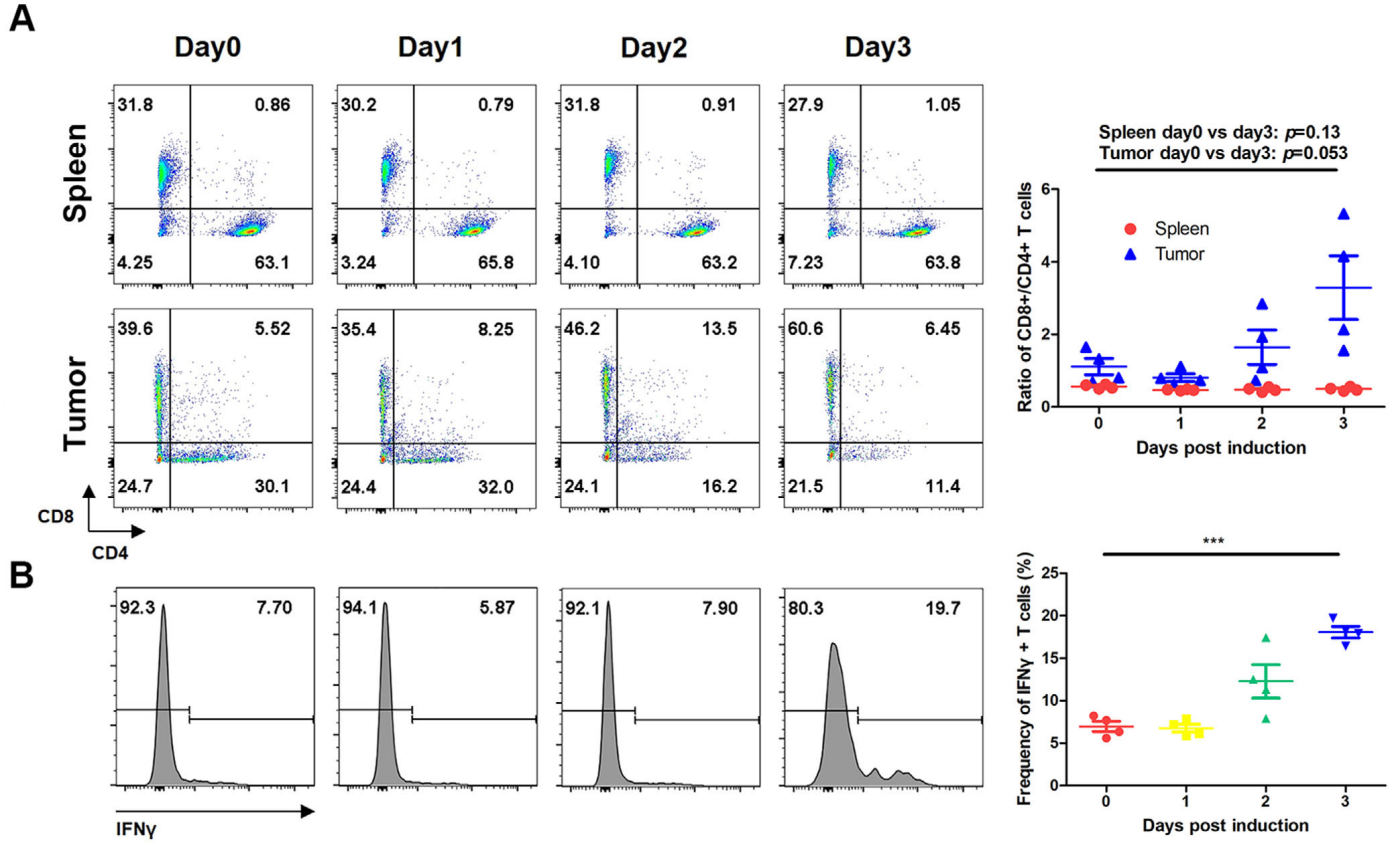
The alterations of T cell status during induced expression of dcIL12IL2GMCSF. The inducible B16 cells were subcutaneously inoculated into the flanks of C57BL/6 mice. At different times after dox administration, splenocytes and tumor‐infiltrating lymphocytes were isolated and subjected to flow cytometry analysis. CD4 and CD8 expressions were analyzed in gated CD3+ cells (A). Graph (B) showed the intracellular staining of IFNγ in CD3+/CD8+ cells from the splenocytes. ***p < 0.001. The experiments were performed twice. *n* = 4

Considering the roles of IL12, IL2, and GMCSF in the immune system, we explored alterations in the immune cells during the antitumor response. Splenocytes and tumor‐infiltrating lymphocytes were collected and subjected to fluorescence‐activated cell sorting (FACS) analysis at different times post‐induction. In the spleen, levels of NK1.1+ and CD3+ subset cells gradually decreased (Figure 2A). However, the CD8+/CD4+ ratios of T cells were not influenced (Figure 3A). In the tumors, levels of NK1.1+ cells reduced, but the levels of CD3+ cells were greatly increased, at day 3 post‐induction (Figure 2A). Notably, the CD8+/CD4+ ratios of T cells were clearly elevated, indicating cytotoxic T cell infiltration in the tumors (Figure 3A). Intracellular interferon gamma (IFNγ) staining demonstrated the presence of activated CD8+ T cells (Figure 3B). CD11c+ cells gradually decreased in the tumors, but increased in the spleens (Figure 2B). However, there were little changes in the levels of CD11c+MHCII+ double positive cells, indicating a variation in other dendritic cell subsets. The changes in the levels of CD11b+ cells were similar to the changes in the CD11c+ population, with both decreasing in the tumors and increasing in the spleens. Moreover, the levels of the CD11b+MHCII+ subset in the spleens were markedly raised post‐induction, suggesting an improvement in the antigen presentation capability of these cells (Figure S1A). There was slight increase in activated B220+ cells in the spleens, and the ratios of these were significantly increased in the tumors (Figure S1B). In addition, we found that the levels of regulatory cell receptor programmed cell death protein 1 (PD1) were greatly unregulated in the T cells (Figures S1C, S1D, and S1E). At day 3 post‐induction, most of the tumor‐infiltrating T cells were PD1 positive, suggesting a powerful negative immune regulation mechanism in their antitumor immunity.

### 2.3 The fusion cytokine scIL12IL2GMCSF exhibited superior immune activation capabilities in vitro and antitumor effects in vivo

**FIGURE 4:**
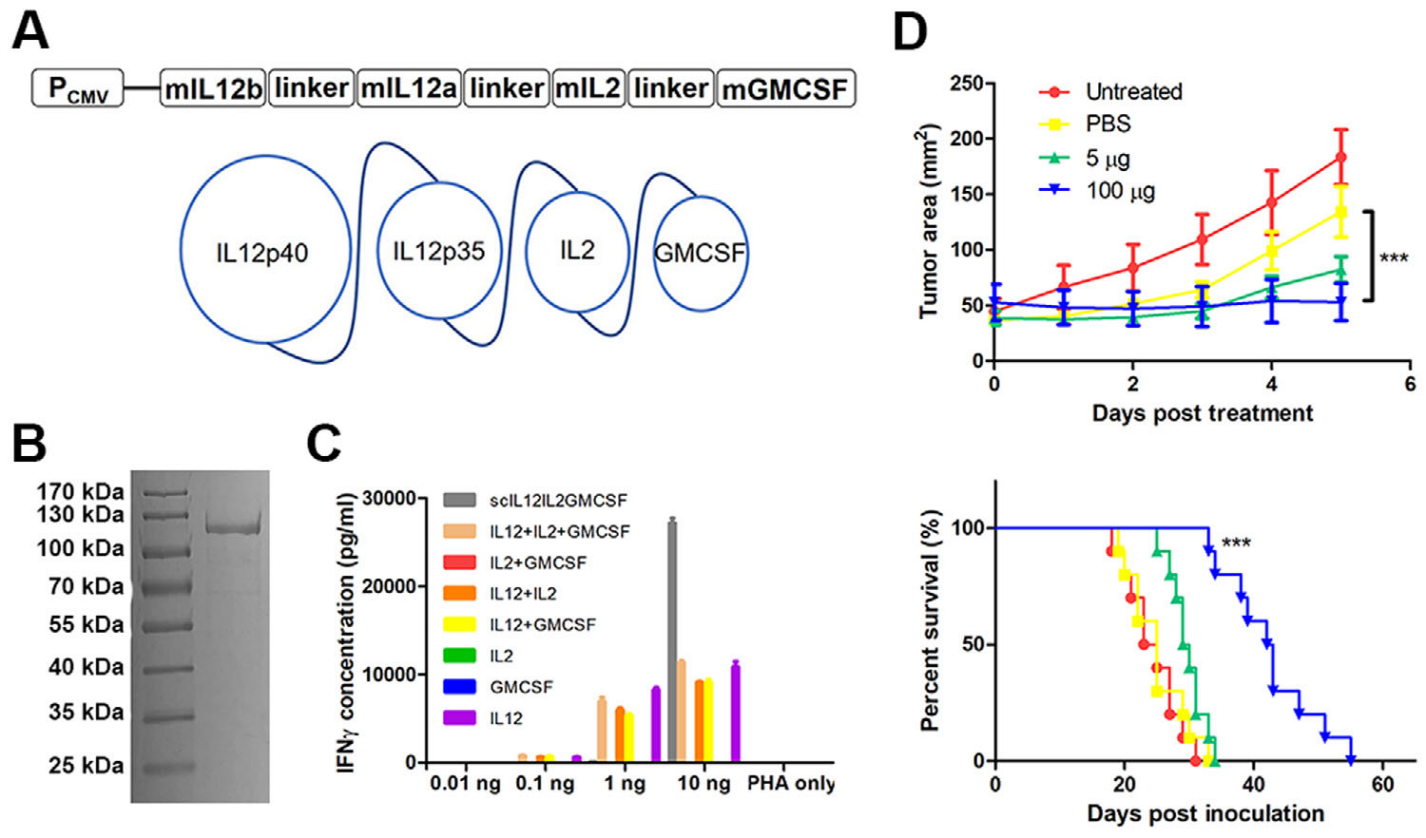
The immune activation capability and antitumor effects of fusion cytokine scIL12IL2GMCSF. (A) Schematic representation of scIL12IL2GMCSF expression cassette and molecule conformation. (B) SDS page electrophoresis of the purified scIL12IL2GMCSF proteins. (C) Splenocytes from the C57BL/6 mice were plated onto 96‐well plates and stimulated with the indicated cytokines plus PHA for 24 h. The levels of IFNγ in the supernatants were measured by ELISA. *n* = 3. (D) B16F10 cells were subcutaneously inoculated into the flank of C57BL/6 mice. Either scIL12IL2GMCSF or PBS was intratumorally injected into the lesions when the tumor diameters reached 5–9 mm. Tumor growth (*n* = 5) and overall survival (*n* = 10) were recorded. ***p < 0.001. These experiments were repeated twice

In previous studies, the expression levels of IL12 from a bi‐cistron construction were found to be low. Thus, we designed a single cistron cassette to improve the productivity of the IL12IL2GMCSF fusion proteins (Figure 4A). ELISA measurement demonstrated that the expression levels of scIL12IL2GMCSF were much higher than that of dcIL12IL2GMCSF, reaching up to 100 μg/ml (Figure S2). Furthermore, the fusion proteins were easily purified using affinity magnetic beads (Figure 4B). Next, the activities of the fusion proteins were detected by stimulating IFNγ secretion in splenocytes. The activation capability of scIL12IL2GMCSF was superior to that of the mixture with equivalent proportions of IL12, IL2, and GMCSF. At low concentrations, low activities from scIL12IL2GMCSF were detected. However, the fusion proteins exhibited higher activity than the other groups at high concentrations (Figure 4C). Subsequently, the therapeutic potential of scIL12IL2GMCSF was evaluated through measurement of the therapeutic effects of intratumoral injections. Considering the influences of solvents on treatments, we compared these effects to the effects of carboxymethyl cellulose, chitosan, and glycerol. The latter two displayed inhibition activities (Figure S3). Glycerol was selected for subsequent study since it is an approved excipient for clinical use. The administration of scIL12IL2GMCSF significantly suppressed subcutaneous B16F10 melanoma growth. The therapeutic outcome was dose‐dependent, and high doses of the protein greatly improved survival rates in the tumor bearing mice (Figure 4D). Some weight loss was observed during treatment, which was unrelated to the drug since injection of PBS alone also produced this effect (Figure S4). Interestingly, administration of low doses of scIL12IL2GMCSF completely eradicated the subcutaneous B16F10‐rtTA tumors (Figure S5). This may be because the rtTA protein provided a predominant neoantigen for effective immune recognition.

### 2.4 Intratumoral injection of scIL12IL2GMCSF induced tumor clearance in various tumor models

**FIGURE 5:**
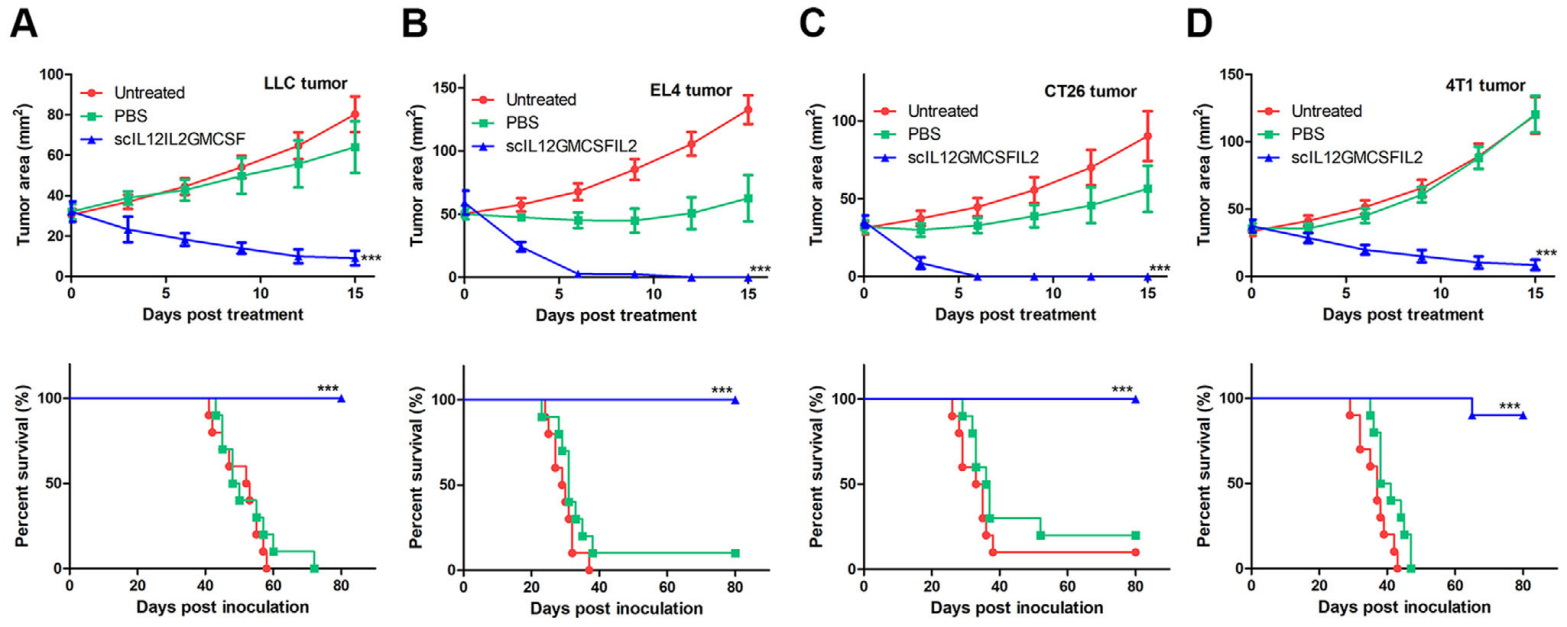
Therapeutic effects of intratumoral administration of scIL12IL2GMCSF in various mouse tumor models. Mouse LLC (A) or EL4 (B) tumor cells were subcutaneously inoculated into the flanks of C57BL/6 mice. Mouse CT26 (C) or 4T1 (D) tumor cells were subcutaneously inoculated into the flanks of BALB/c mice. When the tumor diameters reached 5–9 mm, 100 μg scIL12IL2GMCSF or PBS was intratumorally injected into the lesions. Tumor growth (*n* = 5) and overall survival (*n* = 10) were recorded. ***p < 0.001. These experiments were repeated twice

Based on the reported antitumor activity in melanoma, we additionally tested the curative effects of scIL12IL2GMCSF in several other malignant tumors in mice. For LLC (Figure 5A), EL4 (Figure 5B), and CT26 (Figure 5C) tumors, single injections induced complete tumor regression without recurrence. There were no deaths reported in the scIL12IL2GMCSF treatment group. For the 4T1 tumor (Figure 5D), one mouse died due to tumor relapse despite reduction in the original tumor size after drug injection. In general, intratumoral injection of scIL12IL2GMCSF exhibited curative potential in tested mouse tumor models.

### 2.5 The immunocytokine scIL12IL2DiaNFGMCSF induced IFNγ expression and immune cell alterations

**FIGURE 6:**
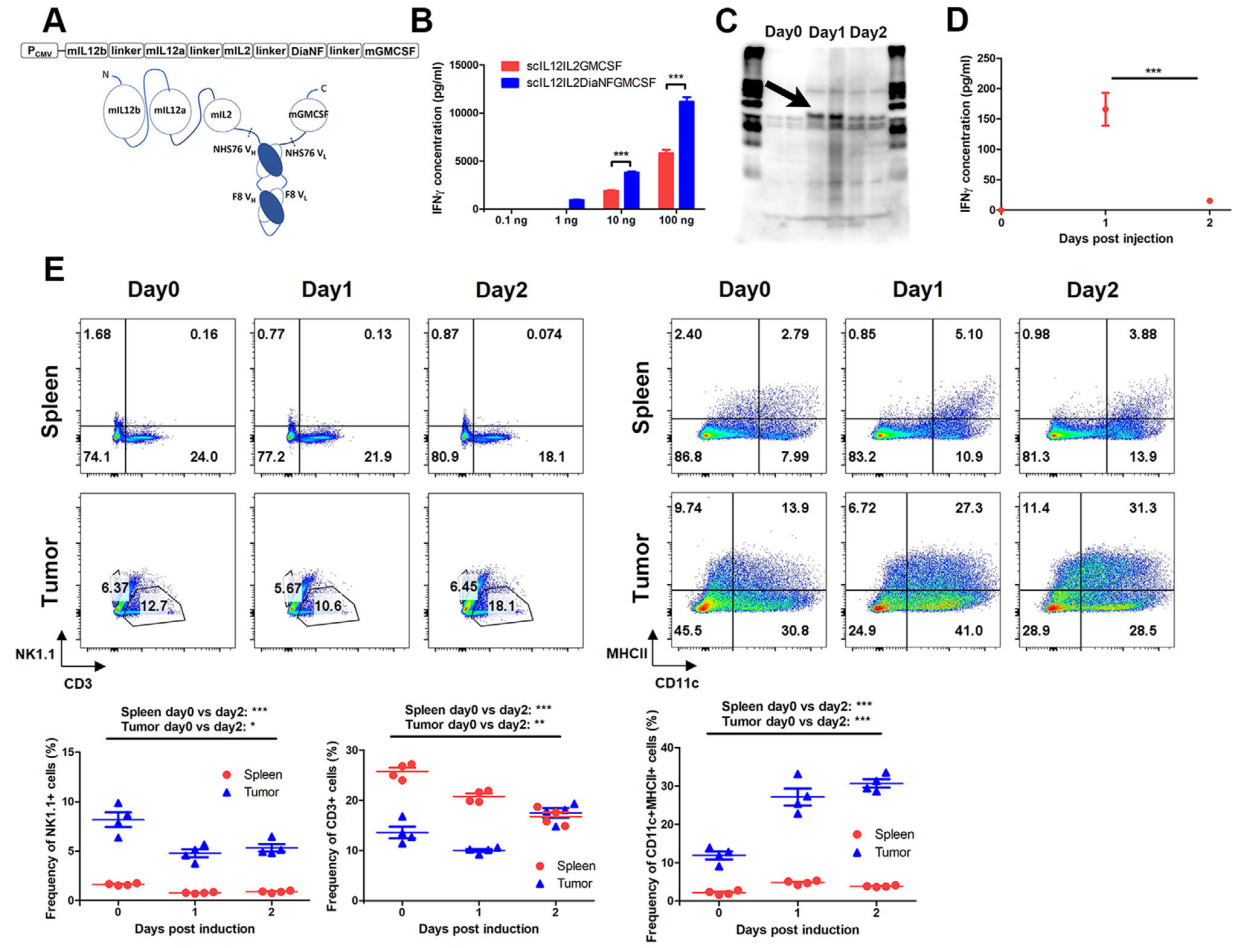
The immunocytokine scIL12IL2DiaNFGMCSF stimulated immune activation in vitro and in vivo. (A) Schematic representation of scIL12IL2DiaNFGMCSF expression cassette and molecule conformation. The dashed lines indicate thrombin cleavage sites. (B) Splenocytes from the C57BL/6 mice were plated onto 96‐well plates and stimulated with scIL12IL2GMCSF or scIL12IL2DiaNFGMCSF plus PHA for 12 h. Levels of IFNγ in the supernatants were measured using ELISA. *n* = 3. (C) Mouse LLC tumor cells were subcutaneously inoculated into the flanks of C57BL/6 mice. When the tumor diameters reached 5–8 mm, 200 μg scIL12IL2DiaNFGMCSF was intravenously injected into mice. Tumor tissues were collected separately at day 0, 1, or 2 after injection and subjected to western blotting analysis, using anti‐IL12p40 antibody. The arrows indicate distinctive bands approximately 75kD in size. (D and E) Mouse LLC tumor cells were subcutaneously inoculated into the flanks of C57BL/6 mice. When the tumor diameters reached 5–8 mm, 200 μg scIL12IL2DiaNFGMCSF was intravenously injected into the mice. At day 0, 1, or 2 after injection, samples of serum were isolated and subjected to ELISA measurement of levels of IFNγ (D), and the splenocytes and tumor‐infiltrating lymphocytes were subjected to flow cytometry analysis (E). The gated CD45+ cells were analyzed with the groups of NK1.1, CD3 or CD11c, MHCII. *p < 0.05; **p < 0.01; ***p < 0.001. The experiments were performed twice. *n* = 4

To improve the translational application of this remedy, we designed an intravenous formulation of the fusion cytokine by adding the tumor‐targeting diabody DiaNF (Figure 6A), which consisted of scFv from F8 and NHS76 antibodies. Measurement of in vitro activity revealed that the IFNγ stimulating capability of scIL12IL2DiaNFGMCSF was higher than that of scIL12IL2GMCSF (Figure 6B). After intravenous injection, the tumor tissues were collected and subjected to western blotting analysis. Compared with the control group, a band of approximately 75 kDa in size was clearly detected in the tumors 1 day post‐injection, which might be an IL12‐IL2 fragment released from scIL12IL2DiaNFGMCSF by thrombin cleavage (Figure 6C). Then, we explored the effects of fusion cytokine administration on the host immune system. After infusion, serum IFNγ levels were clearly elevated but rapidly declined to low levels within 2 days, indicating a transient systemic immune activation (Figure 6D). It also caused some immune cell alterations detected by FACS. There was a significant increase in the levels of tumor‐infiltrating CD3+ cells (Figure 6E), in which the CD8+/CD4+ ratios were greatly raised (Figure S6). The levels of T cells in the spleens were slightly decreased, without changes in the CD8+/CD4+ ratios (Figures 6E and S6). The levels of CD11c+ cells were increased in both the tumors and spleens, and the intratumor cells seemed to be activated due to the enhanced expressions of MHCII molecules (Figure 6E). Although no changes were observed in the spleens, CD11b+ cells were activated in the tumors with elevated MHCII expressions, similar to in CD11c+ cells (Figure S6). These results suggest that intravenous administration of scIL12IL2DiaNFGMCSF efficiently induced immune activation, especially in tumors.

### 2.6 Intravenous administration of scIL12IL2DiaNFGMCSF suppressed tumor growth in various tumor models

**FIGURE 7:**
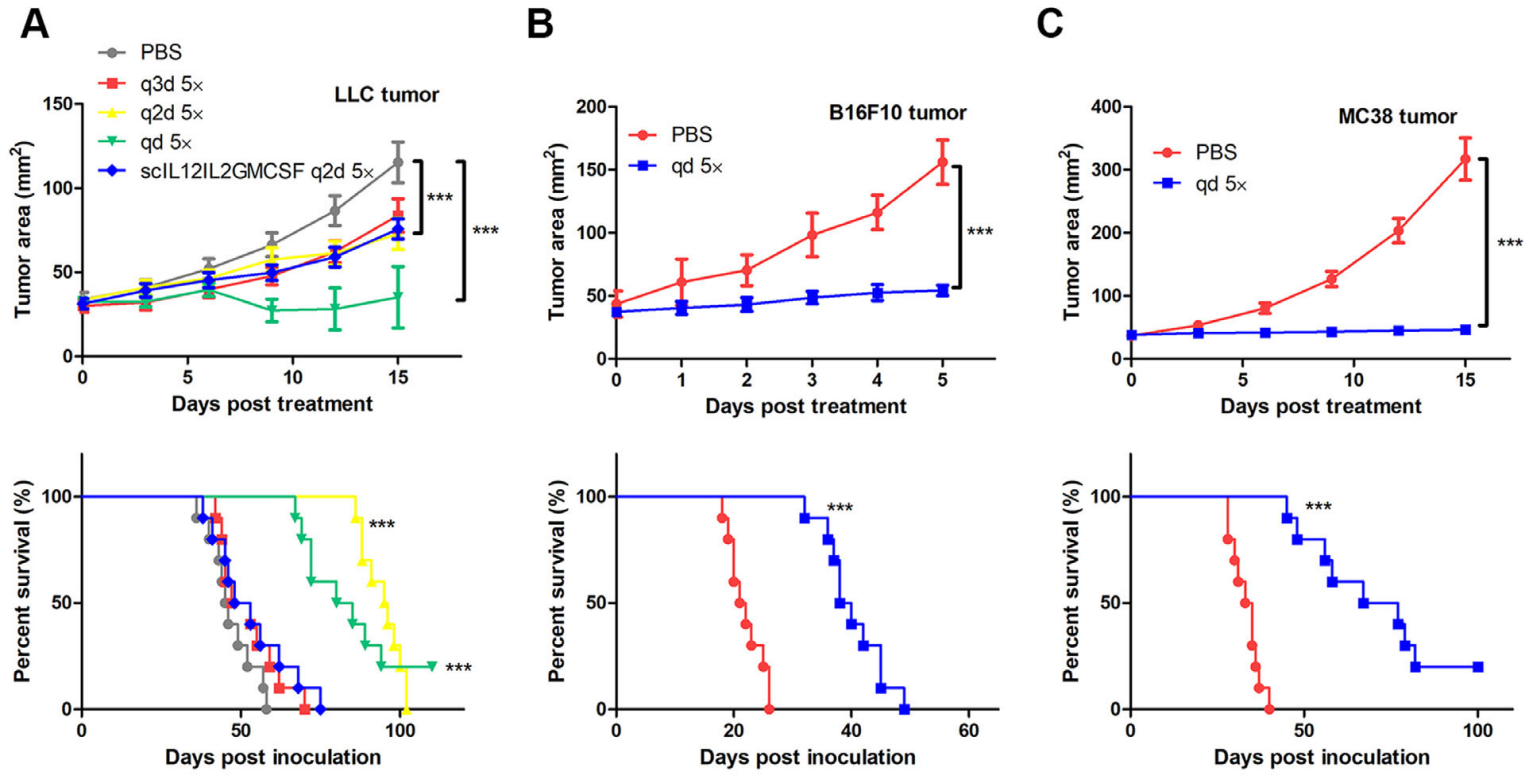
Therapeutic effects of intravenous administration of scIL12IL2DiaNFGMCSF in various mouse tumor models. (A) Mouse LLC tumor cells were subcutaneously inoculated into the flanks of the C57BL/6 mice. When the tumor diameters reached 5–9 mm, 50 μg scIL12IL2DiaNFGMCSF was intravenously injected daily (qd), every 2 days (q2d) or every 3 days (q3d), up to a total five doses. The scIL12IL2GMCSF was intravenously injected every 2 days (scIL12IL2GMCSF q2d). Tumor growth (*n* = 5) and overall survival (*n* = 10) were recorded. Mouse B16F10 (B) or MC38 (C) tumor cells were subcutaneously inoculated into the flanks of C57BL/6 mice. When the tumor diameters reached 5–9 mm, daily intravenous administration of 50 μg scIL12IL2DiaNFGMCSF was conducted for 5 days. Tumor growth (*n* = 5) and overall survival (*n* = 10) were recorded. ***p < 0.001. These experiments were repeated twice

Next, the therapeutic effects of scIL12IL2DiaNFGMCSF were evaluated in mouse tumor models. To identify the best administration schedule, the fusion cytokine was intravenously injected into LLC tumors bearing mice either daily (qd), every 2 days (q2d), or every 3 days (q3d) up to a total five doses. Although there were no significant differences in the survival rates between the qd and q2d groups, the former displayed higher tumor growth suppression (Figure 7A). The mice bearing B16F10 or MC38 tumors were treated using the qd schedule. The intravenous administration greatly suppressed tumor growth and increased survival time (Figures 7B and 7C). Importantly, no weight loss was observed throughout the therapeutic process (Figure S7). This indicates that the antitumor effects of the fusion cytokine can be achieved with systemic administration.

## 3 DISCUSSION

In our previous study, we revealed the presence of antitumor activity in some antitumor cytokine combinations using an in vivo cytokine screening system. Among them, the combination of IL12+GMCSF+IL2 showed the most promising therapeutic potential. However, cytokine combinations are hard to translate into pharmaceuticals due to the expensive cost to manufacture three biomolecules and the difficulty to maintain quality control of the protein mixture. When linked by peptide linkers, the fusion proteins showed comparable antitumor effects. This fusion may ameliorate the toxicity of these cytokines because a reduction in the number of acute deaths was observed in mice bearing large, induced tumors in the fusion protein group. Some IL12‐IL2 fusion cytokines also exhibited good safety in tumor bearing mice.^36^, ^37^ Compared to individual cytokines, one IL12IL2GMCSF fusion molecule can bind to IL12, IL2, or GMCSF receptors, making it much easier to be captured and retained at tumor sites. This also reduces systemic leakage and consequent toxicity. Notably, the yield of two‐chain IL12IL2GMCSF fusions was only at the μg/ml level, which is close to other IL12‐based cytokine fusions,^38^, ^39^ likely attributed to the homodimerization of IL12p40 subunit. The yield of single‐chain IL12IL2GMCSF proteins reaches 100 μg/ml, providing a molecular entity more suitable for large scale manufacture.

The concentration of cytokines at the tumor site should be kept constant to provide sustained immune stimulation. Many slow release materials have been experimented for local cytokine delivery.^40^, ^41^ Poly(lactic‐co‐glycolic acid) microspheres are frequently used to encapsulate cytokines or cytokine expressing cells, while also exhibiting good safety and sustained release characteristics.^42^, ^43^, ^44^ Chitosan and alginate are also widely examined in preclinical studies.^45^, ^46^, ^47^, ^48^ However, these methods are either too complicated to operate or not approved for clinical use. In this work, glycerol is utilized as a carrier to deliver cytokine proteins. First, the viscosity of glycerol allows for retention of drugs at the injection site. Second, glycerol is a common preservative of purified protein, which is beneficial in maintaining the activity of cytokines. Moreover, injection of glycerol alone exhibits certain antitumor effects, which might enhance the therapeutic effects of cytokines through a synergistic effect. Of note, administration of glycerol brings systemic toxicities, such as weight loss in tumor bearing mice. This is likely due to the high dose/weight ratio when administered in mice, which can be adjusted in humans.

Despite discovery many years ago, cytokines are restricted in their clinical application due to their toxicities. In addition to local delivery, antibody conjugated cytokines are also a good choice to reduce the toxicities.^49^, ^50^ Compared to free cytokines, immunocytokines exhibit superior therapeutic effects in various mouse tumors.^51^, ^52^ In a previous research, an immunocytokine carrying an IL12‐IL2 payload markedly suppressed the growth of the Epcam‐LLC tumor, indicating the potential of immunocytokines with multiple cytokine payloads.^37^ Alternatively spliced extra‐domain A (EDA) and extra‐domain B of fibronectin, histone‐DNA complex (HDC), epidermal growth factor receptor (EGFR), et al, are the frequently used targets in these molecule designs. Some IL12‐based immunocytokines have entered clinical studies. Among them, BC1‐IL12 and NHS‐IL12 have been proven safe in stage I clinical trials.^53^, ^54^ The MTDs are 15 μg/kg for the former and 16.8 μg/kg for the latter. Considering that the MTD of free IL12 was reported to be 0.5–1.0 μg/kg in previous clinical studies, the fusion of antibodies to cytokines may greatly ameliorate the toxicities of systemic administration. In this study, we constructed a bispecific scFv targeting HDC and EDA. Anti‐HDC is placed at inner area, since its target is free and less influenced by steric interference. Compared to single target, this design provides an additional chemotactic factor and may improve the targetability of the immunocytokines. In this study, we found that compared to scIL12IL2GMCSF, scIL12IL2DiaNFGMCSF displayed higher immune activation capability through an experiment involving the stimulation of splenocytes in vitro. This might be due to the change in molecule conformation caused by diabody addition, making it easier for IL2 and GMCSF to bind to their receptors on immune cells.

Clinical practice has indicated that monotherapy for cancer, including immunotherapy, reaches a ceiling. Recently, some novel combinational immunotherapies have been found to exhibit excellent curative effects.^55^, ^56^, ^57^, ^58^ These results suggest that multiple immune stimulations have the capability to elicit immune responses that allow for the elimination of malignant tumors. Considering the functions of IL12, IL2, and GMCSF in the immune system, it can be envisaged that a positive feedback loop plays a pivotal role in fusion protein mediated tumor elimination (Figure S8). Briefly, IL12 and IL2 synergistically activate NK cells to kill malignant cells,^59^, ^60^ especially those that escape T cell surveillance through downregulation of MHC class I and β2m. If presence, preexisting infiltrating T cells also participate in the antigen release process. A group of super dendritic cells with enhanced antigen presentation capability, acquired from the synergy of IL12 and GMCSF,^61^, ^62^ captures those tumor antigens and migrates to draining lymph nodes. This allows for priming of tumor‐specific T cells, including clones against neoantigens in low abundance. These T cell clones are further activated and expanded by the synergistic action of IL12 and IL2,^63^, ^64^ which allows for infiltration into tumors to kill more malignant cells. This killing‐presentation cycle then finally generates a multiplex antitumor immune repertoire against most heterogeneous cancer cells. Based on this mechanism, it is reasonable to conclude that mice cured from melanoma may acquire resistance to other unrelated syngeneic tumors. Taken together, our research presents novel fusion proteins that exhibit great antitumor potential and may be translated into clinical application for human cancer treatment.

## 4 MATERIALS AND METHODS

### 4.1 Cell lines and mice

Mouse B16F10 melanoma, LLC, EL4 lymphoma, CT26.WT colon carcinoma, MC38 colon adenocarcinoma, human embryonic kidney cell line 293, and 293FT were cultured in Dulbecco's modified Eagle's Medium (DMEM) supplemented with 10% fetal bovine serum (FBS) (Life Technologies) and penicillin/streptomycin (Life Technologies) at 37℃ in 5% CO2 incubator. Mouse 4T1 mammary carcinoma cell line was cultured in RPMI1640 medium supplemented with 10% FBS (Life Technologies) and penicillin/streptomycin (Life Technologies) at 37℃ with 5% CO2. The cell lines were obtained from the Laboratory of Antigen Presentation, Institute of Immunology, Third Military Medical University on September, 2017. No testing was done since that time. The passage number of cells used in this study was less than 30.

C57BL/6 and BALB/c mice were purchased from Beijing Huafukang Bioscience Company. Mice that were 8–16 weeks old were used for the tumor experiments. All animals were raised under normal environmental conditions. The animal studies were conducted in accordance with guidelines for the care and use of laboratory animals, and with the approval of the Institute Animal Care and Use Committee of Tsinghua University.

### 4.2 Plasmid construction and cell transduction

The construction of an inducible expression system was described in a previous study.^35^ The expression of target cistrons can be induced by the addition of doxycycline (dox). The dcIL12IL2GMCSF is a fusion protein consisting of two cistrons, IL12b‐GMCSF and IL12a‐IL2, ligated by a T2A peptide (Figure 1A). The DNA encoding dcIL12IL2GMCSF was synthesized and subcloned into a pLentis‐TRE‐MCS‐PGK‐PURO vector between the BamHI and XhoI sites, generating the inducible vector pLentis‐TRE‐dcIL12IL2GMCSF‐PGK‐PURO. The scIL12IL2GMCSF is a fusion protein in which IL12b, IL12a, IL2, and GMCSF were tandemly ligated by (G_4_S)_3_ linkers (Figure 3A). The scIL12IL2DiaNFGMCSF was designed by inserting the heterogenous tumor targeting diabody DiaNF between the IL2 and GMCSF of scIL12IL2GMCSF (Figure 5A). The thrombin cleavage sequence LVPRGS was inserted into the linkers between the DiaNF and cytokines. The DNA encoding dcIL12IL2GMCSF, scIL12IL2GMCSF, and scIL12IL2DiaGMCSF were synthesized and subcloned into the vector pLentis‐CMV‐MCS‐IRES‐PURO between the BamHI and XhoI sites, generating the expression vectors pLentis‐CMV‐dcIL12IL2GMCSF‐IRES‐PURO, pLentis‐CMV‐scIL12IL2GMCSF‐IRES‐PURO, and pLentis‐CMV‐scIL12IL2DiaNFGMCSF‐IRES‐PURO. Addition of 6*His to the C terminal of fusion proteins was performed to provide an affinity purification tag. The constructed plasmids are summarized in Figure S9.

The lentiviral particles of these vectors were produced by cotransfection of pMD2.G, psPAX2, and lentiviral vectors into 293FT cells. B16F10‐rtTA cells were transduced with pLentis‐TRE‐dcIL12IL2GMCSF‐PGK‐PURO virus and selected with 3 μg/ml puromycin plus 8 μg/ml blasticidin, generating doxycycline inducible cells, B16F10‐rtTA(TRE‐dcIL12IL2GMCSF). For protein expression, 293 cells were transduced with pLentis‐CMV‐dcIL12IL2GMCSF‐IRES‐PURO, pLentis‐CMV‐scIL12IL2GMCSF‐IRES‐PURO, and pLentis‐CMV‐scIL12IL2DiaNFGMCSF‐IRES‐PURO virus and selected using 3 μg/ml puromycin, generating the stable transduced cells, 293(dcIL12IL2GMCSF), 293(scIL12IL2GMCSF), and 293(scIL12IL2DiaNFGMCSF).

### 4.3 Induction of expression of dcIL12IL2GMCSF

B16F10‐rtTA(TRE‐dcIL12IL2GMCSF) cells were plated onto 24 well plates at 5 × 10^4^ cells/well in 700 μl of medium. Separate administration of 100 ng/ml dox was done at 24, 48, or 72 h, and all supernatants were collected at 96 h post cell plating. The concentrations of fusion protein in the supernatants were measured with mouse IL12p70 ELISA Kits (Neobioscience) according to the manufacturer's instructions.

A total of 10^5^ B16F10‐rtTA(TRE‐dcIL12IL2GMCSF) cells were subcutaneously injected into the right flanks of the mice. When the tumors reached the indicated size, dox was administered by adding 2 g/L dox into the drinking water of the mice. The perpendicular diameters of tumors were measured using a caliper, and the tumor areas were calculated by using the equation: long diameter×short diameter. To explore the antitumor immune memory, 10^5^ B16F10, 2×10^5^ LLC, or 10^6^ EL4 tumor cells were subcutaneously injected into the left flanks of the cured mice 10 months after primary tumor eradication.

### 4.4 Flow cytometric analysis of the immune cells

Mouse spleens were minced by a syringe plunger on a 70 μm cell strainer. After lysis of red blood cells, the dissociated splenocytes were rinsed with phosphate buffered saline (PBS) and resuspended in FACS buffer (PBS+2%FBS+5mM EDTA). Harvested solid tumor tissues were dissociated into single cells by mechanical disaggregation followed by collagenase digestion (DNase 1 μg/ml, collagenase type II 1 mg/ml, CaCl_2_ 5 mM in PBS) at 37℃ for 1 h. After passing through the 70 μm cell strainer, the tumor infiltrating lymphocytes (TIL) were isolated using 40:80% Percoll (GE healthcare). Prior to staining, the splenocytes and TILs were blocked for 30 min in FACS buffer containing Fc Blocker (1 μg/ml). The cells were stained with the following antibodies: mouse CD45 AF700, mouse CD3 BV421, mouse CD4 PE, mouse CD8 FITC, mouse NK1.1 APC, mouse PD1 PE/CY7, mouse CD11B APC, mouse CD11C PE, mouse MHCII FITC, mouse B220 BV510, mouse mIgD PE/CY7 and mouse IFNγ APC/CY7 (Biolegend 109821, 100335, 100407, 100705, 108710, 135215, 101211, 117307, 109905, 103247, 405719, 505849). FVD506 (Ebioscience 650866) and 7AAD (Biolegend 420403) were used to distinguish viable cells. Cells were detected by flow cytometry using a BD LSRFortessa Flow cytometer. Acquired data were analyzed using Flowjo software.

### 4.5 Production of recombinant proteins

The 293(dcIL12IL2GMCSF), 293(scIL12IL2GMCSF), and 293(scIL12IL2DiaNFGMCSF) cells were separately inoculated onto 15 cm dishes in DMEM medium with 10% FBS. After the confluences reached 90%, the culture mediums were replaced with 35 ml CDM4HEK293 medium (Hyclone) supplemented with 4 mM L‐Glutamine. After 5 days, the supernatants were collected and filtrated through a 0.45 μm filter (Millipore). After concentrating the cytokine solutions to 1 ml using Amicon Ultra‐15 centrifugal filter unit (Millipore), they were purified using a BeaverBeads IDA‐Nickel Kit (Beaverbio) according to the manufacturer's instructions. The cytokine concentrations were determined using mouse IL12p70 ELISA Kits. The purified cytokine solutions were aliquoted and stored at −20°C. The purified scIL12IL2GMCSF proteins were sampled and diluted to 50 ng/μl, then subjected to SDS page electrophoresis under reduced conditions.

### 4.6 Measurement of fusion cytokine bioactivity

Splenocytes from the C57BL/6 mice were resuspended in RPMI1640 (2% FBS) with PHA (Ebioscience 00497703) plus indicated cytokines. The IL12, IL2, and GMCSF were purchased from Peprotech. Cells were plated onto 96 well plates (6 × 10^5^/well for experiments in Figure 4 and 2 × 10^5^/well for experiments in Figure 6) and cultured for 24 h. The supernatants were collected, and their levels of IFNγ were measured using the Mouse IFN‐gamma Quantikine ELISA Kit (R&D systems).

### 4.7 Comparison of carboxymethyl cellulose, chitosan, and glycerol

A total of 10^5^ B16F10 cells were subcutaneously injected into the right flanks of mice. The treatment was initiated when the tumor diameters reached around 5 mm. An amount of 5 μg scIL12IL2GMCSF protein was mixed separately with 50 μl 1% carboxymethyl cellulose, 50 μl 3% chitosan (chitosan glutamate, Protosan G 213, NovaMatrix), or 60 μl glycerol, totaling to a 100 μl intratumoral injection. The tumor sizes were monitored daily after the intratumoral administration.

### 4.8 Tumor treatment

A total of 10^5^ B16F10, 10^5^ B16F10‐rtTA, 2×10^5^ LLC, 10^6^ EL4, or 5×10^5^ MC38 cells were subcutaneously injected into the right flanks of C57BL/6 mice. Moreover, a total of 10^6^ 4T1 or 10^5^ CT26 cells were subcutaneously injected into the right flanks of BALB/c mice. The treatments were initiated when the tumor diameters reached 5–9 mm.

For the intratumoral administrations, the cytokine solutions were diluted to 40 μl with PBS and mixed with 60 μl glycerol prior to injection. The glycerol/cytokine solution was slowly injected into tumors using a 29G insulin syringe while avoiding bubbling. In the 4T1 tumor treatment, additional injections were conducted as tumor size increased or as new nodules appeared.

For the intravenous administrations, 50 μg recombinant protein was diluted to 200 μl with PBS prior to injection. Five consecutive injections were conducted daily (qd), every 2 days (q2d), or every 3 days (q3d). Injection of PBS daily was used as control.

Tumor sizes and mouse weight were recorded after treatment.

### 4.9 Western blotting

A total of 2 × 10^5^ LLC cells were subcutaneously injected into the right flanks of C57BL/6 mice. An amount of 200 μg scIL12IL2DiaGMCSF in 200 μl PBS was intravenously injected when the tumor diameters grew to 5–8 mm. The mice were sacrificed at 0, 1, or 2 days after injection. The total proteins from the tumor tissues were extracted using radio immunoprecipitation assay (RIPA). Western blotting was then carried out following standard procedure and using the rabbit antibody anti‐IL12p40 (Abcam).

### 4.10 Immune activation measurement after intravenous administration of recombinant protein

A total of 2 × 10^5^ LLC cells were subcutaneously injected into the right flanks of C57BL/6 mice. An amount of 200 μg scIL12IL2DiaGMCSF in 200 μl PBS was intravenously injected when the tumor diameter grew to 5–8 mm. The mice were sacrificed at 0, 1, or 2 days after injection. The serum IFNγ levels were detected using a Mouse IFN‐gamma Quantikine ELISA Kit (R&D systems), and splenocytes were subjected to FACS analysis of their immune cell populations.

### 4.11 Statistics

Statistical analysis was carried out using GraphPad Prism 5 software. Survival curves were analyzed using the log‐rank (Mantel‐Cox) test. The comparison of growth curves was conducted with two‐way ANOVA. A value of *p* < 0.05 was considered to indicate statistical significance.

## CONFLICT OF INTEREST

Jinyu Zhang has patents CN201910348357, CN201910351987, and CN201910685007 pending. The other author declares no conflict of interests.

## ETHICS STATEMENT

The animal studies were approved by the Institute Animal Care and Use Committee of Tsinghua University.

## AUTHOR CONTRIBUTIONS

Jinyu Zhang designed the study, carried out most of the experiments and prepared the manuscript. Xuan Zhao performezd experiments including immune cell flow cytometry detection, in vitro activity measurement of the fusion proteins, western blot, and serum IFNγ measurement.

## Supporting information

Supporting Information

## Data Availability

The data that support the findings of the study are available from the corresponding author upon reasonable request.
