# Supplementary material for: Administration of fusion cytokines induces tumor regression and systemic antitumor immunity
Source: MedComm (2020). 2021 May 4;2(2):256–68. doi: 10.1002/mco2.68 (PMC8491205; doi:10.1002/mco2.68)
Supplement: Supplementary file 1 — Supporting Information [file MCO2-2-256-s001.pdf]

Administration of fusion cytokines induces tumor regression and systemic antitumor immunity

Authors: Jinyu Zhang[1]*, Xuan Zhao[2]

Affiliations:

[1] Mianyi Biotech Corporation, Chongqing 401332, China.

[2] Institute for Immunology and School of Medicine, Tsinghua University, Beijing 100084, China.

*To whom correspondence should be addressed:

Jinyu Zhang

Mianyi Biotech Corporation

Xiyong Road, Shapingba district

Chongqing, 401332, China

Phone: 86-23-65805875

Email: zhangjinyu@tsinghua.org.cn

Supplementary Materials

Figure S1 The alterations in lymphocytes during induced expression of dcIL12IL2GMCSF.

Figure S2 Expression levels of dcIL12IL2GMCSF and scIL12IL2GMCSF.

Figure S3 Comparison of the therapeutic effects of scIL12IL2GMCSF in different solvents.

Figure S4 Mouse body weight changes during the treatment of B16F10 by intratumoral injection.

Figure S5 Therapeutic effects of scIL12IL2GMCSF in B16F10-rtTA tumor.

Figure S6 The alterations in lymphocytes after intravenous injection of scIL12IL2DiaNFGMCSF.

Figure S7 Mouse body weight changes during the treatment of B16F10 by intravenous injection.

Figure S8 Speculative mechanism of the fusion cytokines in antitumor immunity.

Figure S9 Schematic diagram of the constructed fusion proteins related plasmids in this study.

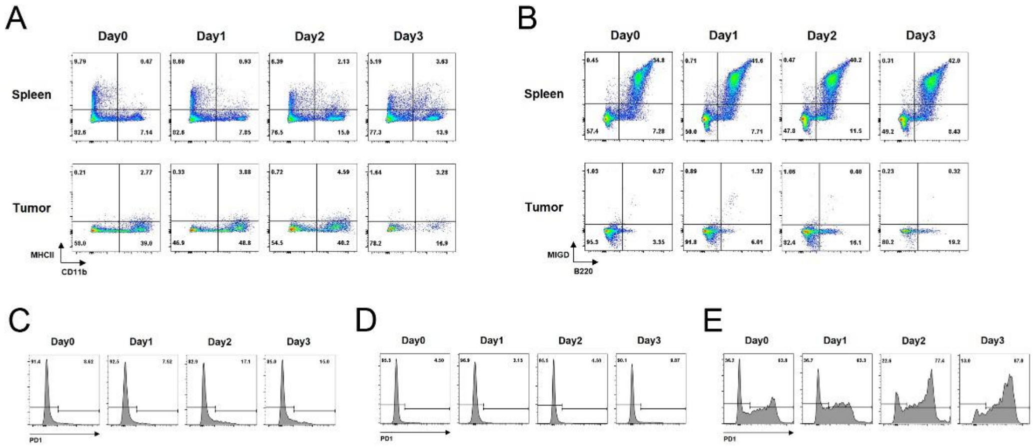

Figure S1 The alterations in lymphocytes during induced expression of dcIL12IL2GMCSF. The inducible B16 cells were subcutaneously inoculated into the flanks of C57BL/6 mice. At different times after dox administration, splenocytes and tumor infiltrating lymphocytes were isolated and subjected to flow cytometry analysis. The gated CD45+ cells were analyzed with the groups of CD11b, MHCII (A) or B220, MIGD (B). The PD-1 expression in spleen CD3+/CD4+ (C), CD3+/CD8+ (D) and tumor-infiltrating CD3+ cells (E) was detected. The experiments were performed twice. n=4.

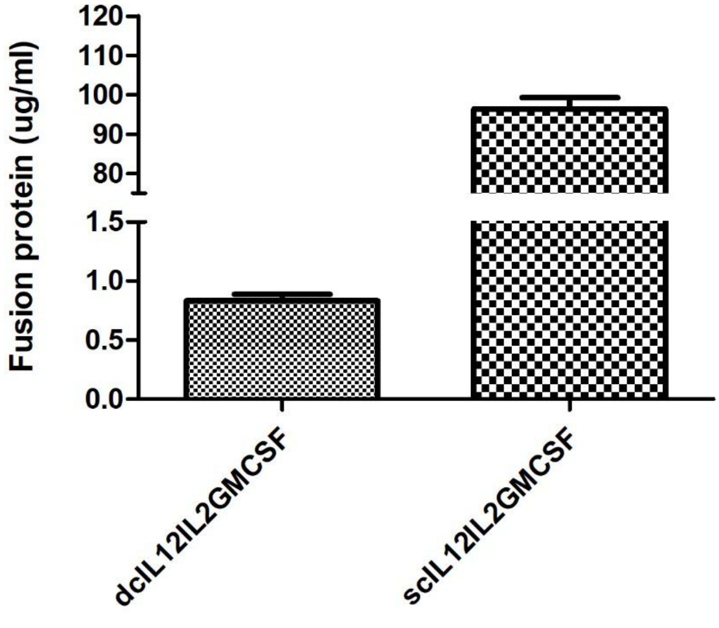

Figure S2 Expression levels of dcIL12IL2GMCSF and scIL12IL2GMCSF. The supernatants of 293(dcIL12IL2GMCSF) or 293(scIL12IL2GMCSF) in CDM4HEK293 culture were collected and subjected to ELISA measurement of the recombinant protein levels using mouse IL12p70 ELISA Kits. n=3.

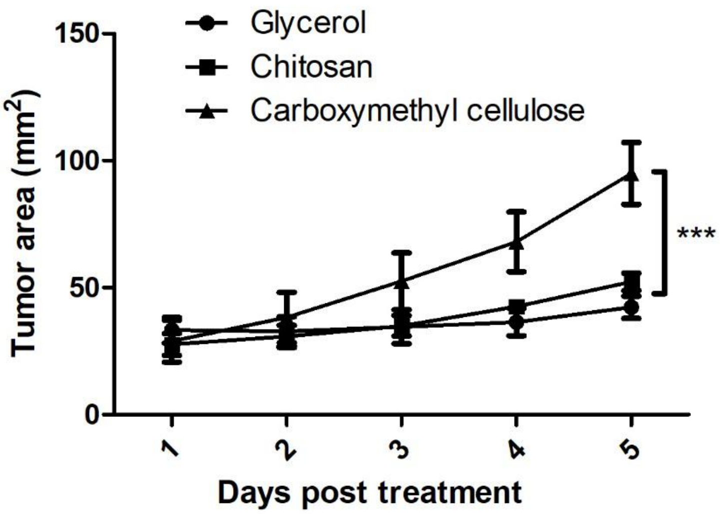

Figure S3 Comparison of the therapeutic effects of scIL12IL2GMCSF in different solvents. B16F10 cells were subcutaneously inoculated into the flanks of the C57BL/6 mice. When the tumor diameters reached ~5 mm, 5 µg scIL12IL2GMCSF in 100 µl 0.5% carboxymethyl cellulose, 1.5% chitosan, or 60% glycerol was intratumorally injected. Tumor growth was recorded daily. n=3, ****p*<0.001. The experiments were repeated twice.

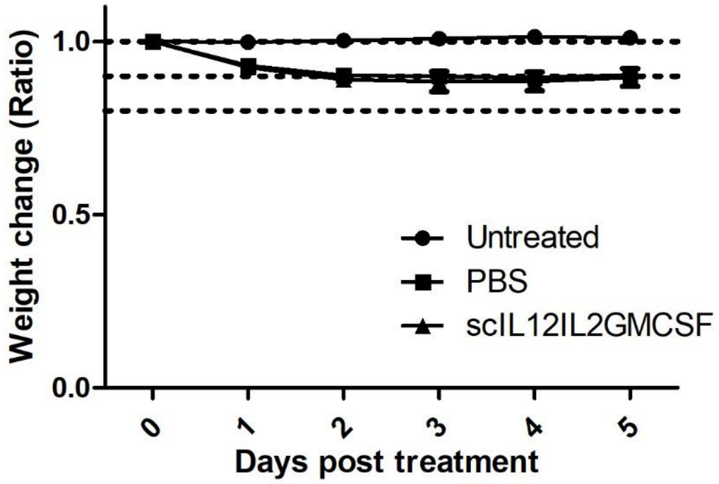

Figure S4 Mouse body weight changes during the treatment of B16F10 by intratumoral injection. B16F10 cells were subcutaneously inoculated into the flanks of C57BL/6 mice. Either scIL12IL2GMCSF or PBS in glycerol solution was intratumorally injected into the lesions when the tumor diameters reached 5-9 mm. Body weights were recorded daily and the change percentages were calculated. Untreated tumor bearing mice were used as control. n=5. The experiments were repeated twice.

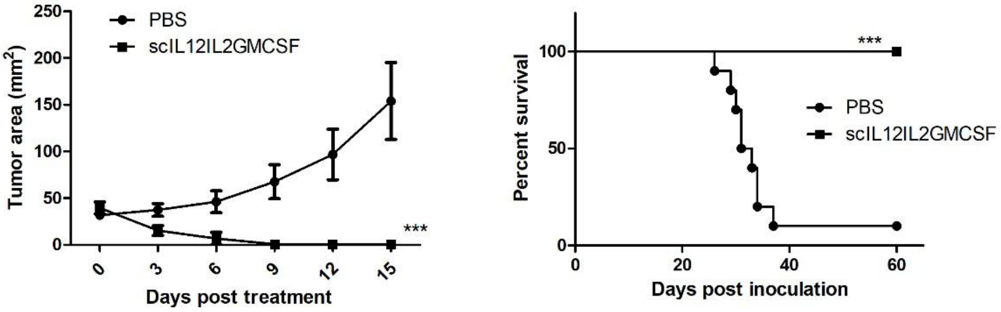

Figure S5 Therapeutic effects of scIL12IL2GMCSF in B16F10-rtTA tumor. B16F10-rtTA cells were subcutaneously inoculated into the flanks of C57BL/6 mice. Either scIL12IL2GMCSF or PBS in glycerol solution was intratumorally injected into the lesions when the tumor diameters reached 5-9 mm. Tumor growth (n=5) and overall survival (n=10) were recorded. ***p<0.001.

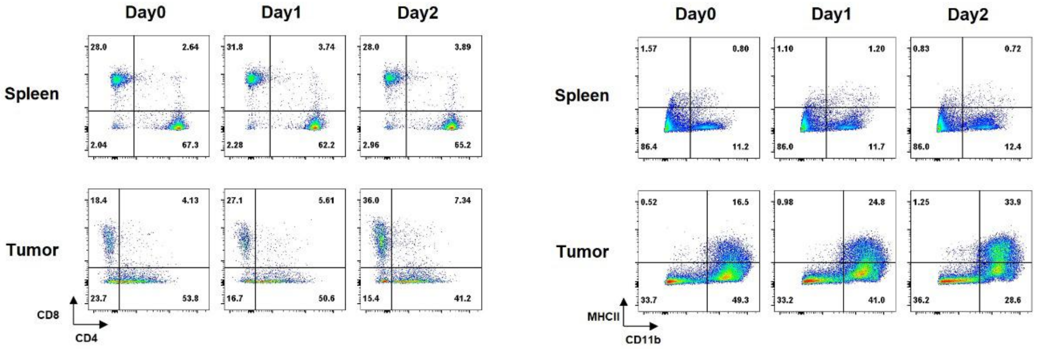

Figure S6 The alterations in lymphocytes after intravenous injection of scIL12IL2DiaNFGMCSF. Mouse LLC tumor cells were subcutaneously inoculated into the flank of C57BL/6 mice. When the tumor diameters reached 5-8 mm, 200 μg scIL12IL2DiaNFGMCSF was intravenously injected into mice. At day 0, 1, or 2 after injection, the splenocytes and tumor infiltrating lymphocytes were subjected to flow cytometry analysis. In gated CD45+ cells, CD11b, MHCII staining group and CD4, the CD8 expression in CD3+ cells were shown. The experiments were performed twice. n=4.

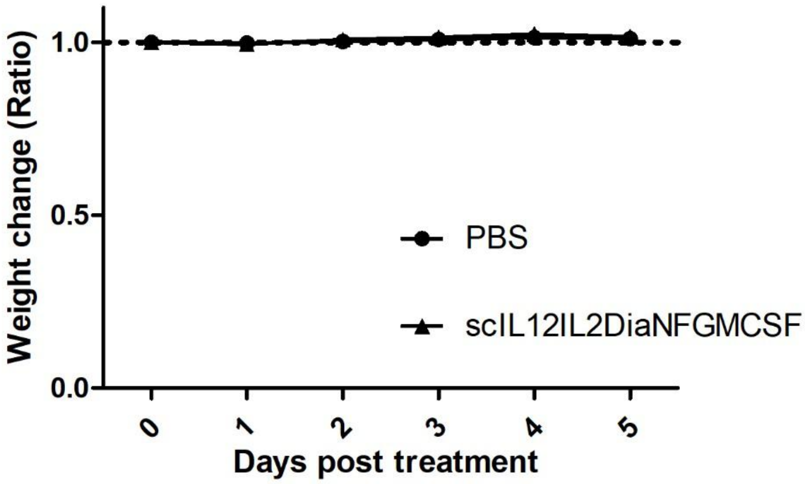

Figure S7 Mouse body weight changes during the treatment of B16F10 by intravenous injection. B16F10 cells were subcutaneously inoculated into the flanks of C57BL/6 mice. When the tumor diameters reached 5-9 mm, daily intravenous administration of 50 μg scIL12IL2DiaNFGMCSF or PBS was conducted for 5 days. Body weights were daily recorded and the change percentages were calculated. n=5. The experiments were repeated twice.

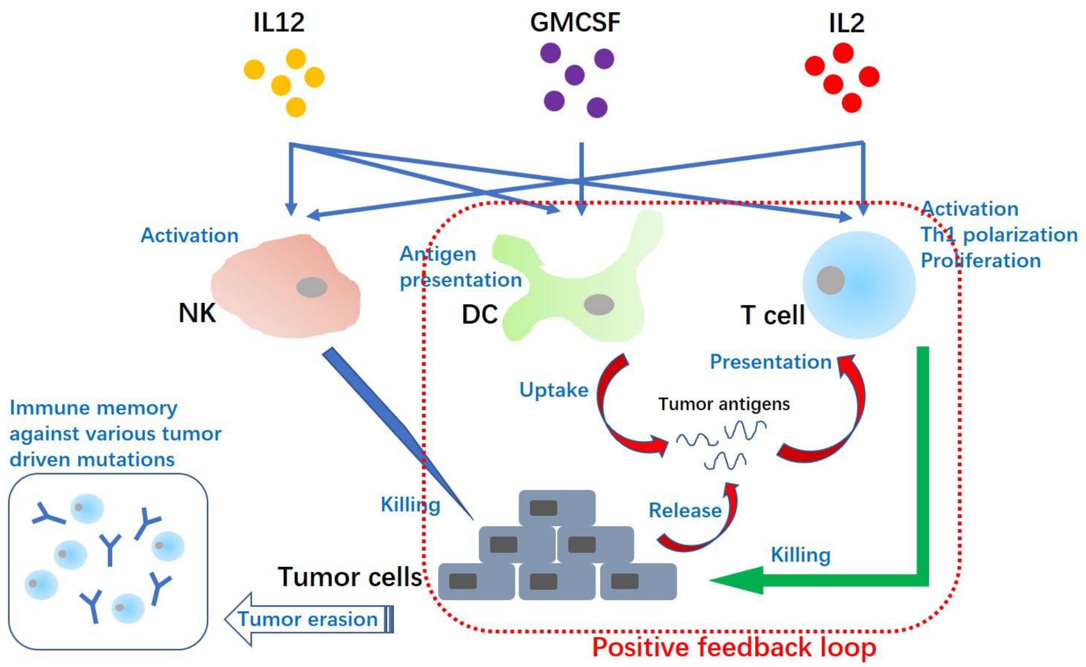

Figure S8 Speculative mechanism of the fusion cytokines in antitumor immunity.

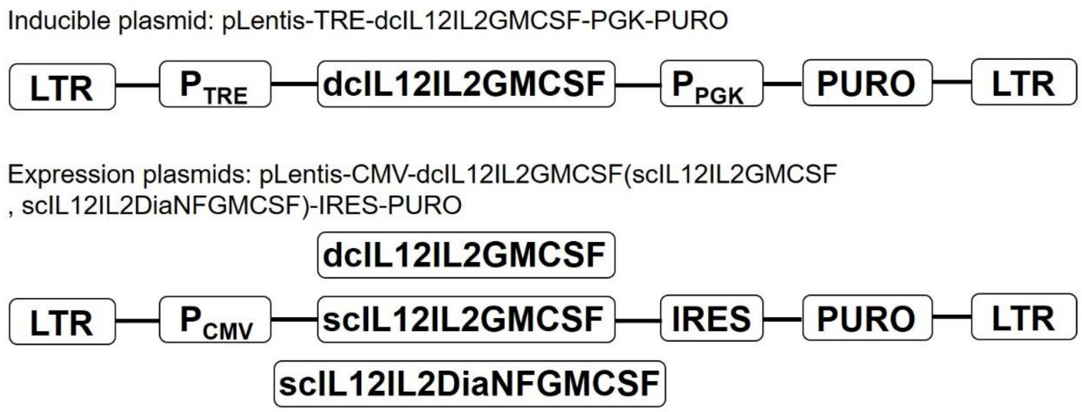

Figure S9 Schematic diagram of the constructed fusion proteins related plasmids in this study.
